# Effectiveness of internal fixation versus arthrodesis on posttraumatic metatarsal-cuneiform joint dislocation: A systematic review and meta-analysis

**DOI:** 10.1051/sicotj/2026041

**Published:** 2026-08-19

**Authors:** Rudiansyah Harahap, Ersa Agriana Novitri, Erviannisa Destrinandia Harahap

**Affiliations:** 1 Ass. Prof. Orthopaedic Department Medical Faculty Muhammadiyah Semarang University Semarang Indonesia; 2 Adhyatma MPH Hospital Semarang Indonesia; 3 Bachelor of Medicine, Faculty of Medicine, Muhammadiyah Semarang University Semarang Indonesia; 4 General Practitioner

**Keywords:** Lisfranc injury, Metatarsal-cuneiform dislocation, Internal fixation, Arthrodesis, Midfoot instability

## Abstract

*Background*: The metatarsal–cuneiform joint, part of the Lisfranc complex, is essential for midfoot stability, load distribution, and gait mechanics. Injuries, often from high-energy trauma such as traffic accidents or falls, can lead to chronic instability, pain, and functional impairment. Despite accounting for only 0.2% of fractures, Lisfranc injuries are frequently missed in initial diagnosis. Surgical management typically involves internal fixation (IF) to preserve mobility or arthrodesis for permanent stability, yet evidence on their long-term effectiveness remains conflicting, highlighting the need for comparative evaluation. *Methods*: A systematic review and meta-analysis were conducted according to PRISMA guidelines. PubMed, Scopus, Medline, and Cochrane Library were searched for studies published from January 2015 to June 2025. Ten studies met the inclusion criteria, comprising 441 patients (284 IF, 157 arthrodesis). Primary outcomes were the American Orthopaedic Foot and Ankle Society (AOFAS) score and Visual Analog Scale (VAS) pain score; secondary outcomes included recovery time and reoperation rate. Data were pooled using mean differences (MD) and percentages with corresponding p-values. *Results*: Arthrodesis yielded significantly higher AOFAS scores compared with IF (85.40 ± 5.51 vs 83.96 ± 5.67; MD = +1.44; *p* = 0.001) and lower VAS pain scores (1.31 ± 1.2 vs 2.56 ± 1.5; MD = −1.26; *p* = 0.038). Mean recovery time was shorter in the arthrodesis group (14.23 ± 4.76 weeks) versus IF (16.34 ± 7.81 weeks; MD = −2.11 weeks; *p* = 0.042). Reoperation rates were significantly lower with arthrodesis (13% vs 30.48%; *p* = 0.031). *Conclusions*: Arthrodesis offers superior functional recovery, pain relief, faster return to ambulation, and fewer reoperations than IF in posttraumatic Lisfranc injuries. Surgical choice should be individualized – arthrodesis is recommended for severe instability requiring maximal stability, while IF may be appropriate for younger or athletic patients prioritizing joint mobility. *Level of Evidence*: Level I, systematic review and meta-analysis.

## Introduction

The metatarsal-cuneiform joint is part of the structure of the Lisfranc joint in the midfoot, which plays an important role in stability, load support, and walking mechanics. Injuries or dislocations in this area often lead to complex complications in the form of joint instability, long-term pain, and long-term functional impairment. This is especially the case due to high-energy direct trauma, such as a traffic accident or falling from a height [1].

The Lisfranc joint is an important articular complex in the midfoot that maintains the stability of the arch of the foot and connects the front foot with the midfoot. This joint consists of five tarsometatarsal joints that are specifically stabilized into a cuneiform mortise by a second osseous metatarsal locking, which is strongly supported by a ligament system, specifically the interosseous Lisfranc ligament. In this complex, post-traumatic dislocation injuries can damage bone elements and ligaments. Damage to the Lisfranc ligament is one of the signs of instability.

Lisfranc injuries account for about 0.2% of all fracture cases with an estimated annual incidence of 1 case per 55,000 people. The trauma mechanism can be differentiated into high-energy trauma such as motor vehicle accidents or falls from heights that dominate severe cases, and low-energy trauma such as sports injuries (soccer, ballet) that are more common in athletes. Demographically, this condition is more prevalent in men with a ratio of 4:1 and most commonly occurs in the age group of 30-40 years. Alarmingly, 20–50% of cases are often missed in the initial diagnosis due to atypical symptoms and their resemblance to a common sprain [2].

One of the main challenges in managing this injury is the selection of the right surgical technique, which is between internal fixation (IF) and arthrodesis. Internal fixation aims to keep the joint in an anatomical position using tools such as screws or plates, and is preferable if you want to maintain joint mobility. On the other hand, arthrodesis (fusion of the joints) is used to eliminate movement and reduce pain, especially in patients at high risk for post-traumatic arthritis [3].

However, whether these two methods are effective in the long run is still a debate. Some studies have shown that arthrodesis produces better clinical outcomes with a reduced risk of pain and smaller recurrence. In contrast, internal fixation can reduce invasion of the joint but is often accompanied by complications such as residual instability and the need for resurgery [4].

This indicates that the choice of surgical technique should be adjusted to the type of dislocation, soft tissue condition, and postoperative risk. Therefore, a comparative clinical evaluation is needed to determine which methods are more effective in accelerating recovery, preventing long-term pain, and maintaining the biomechanical function of the leg [5].

This study aims to analyze the effectiveness of the Internal Fixation and Arthrodesis surgical approach on Post-traumatic Metatarsal-Cuneiform Joint dislocation based on to evaluate the functional outcomes (recovery time and AOFAS score) of patients after internal fixation and arthrodesis procedures, analyze the level of pain (VAS score), and analyze the time of postoperative regeneration and reoperative.

## Method

This report is based on the PRISMA guidelines. PRISMA (Preferred Reporting Items for Systematic Reviews and Meta-Analyses) is an international guide used to compile and report systematic reviews and meta-analyses in a transparent, complete, and structured manner.

Two independent reviewers screened all retrieved titles, abstracts, and full-text articles according to the predefined inclusion and exclusion criteria. Any disagreements regarding study eligibility were resolved through discussion and consensus, and when necessary, a third reviewer was consulted. Data extraction was also independently performed by two reviewers using a standardized data collection form. Extracted variables included author, publication year, study design, country, sample size, patient demographics, type of intervention, duration of follow-up, and reported clinical outcomes.

The methodological quality of included studies was assessed independently by two reviewers. Randomized controlled trials were evaluated using the Cochrane Risk of Bias 2 (RoB 2) tool, while observational cohort and retrospective studies were assessed using the Newcastle Ottawa Scale (NOS). Any discrepancies were resolved by consensus. The results of the quality assessment were considered during interpretation of pooled findings.

Meta-analysis was performed using Review Manager (RevMan) software. Continuous variables including AOFAS score, VAS pain score, and recovery time were pooled using mean differences (MDs) with 95% confidence intervals (CIs). Dichotomous outcomes such as reoperation rates were analyzed using risk ratios (RRs) with 95% CIs. Statistical heterogeneity was assessed using Cochran’s Q test and the *I*^2^ statistic. An *I*^2^ value >50% indicated substantial heterogeneity, and a random-effects model was applied; otherwise, a fixed-effect model was used. A *p*-value <0.05 was considered statistically significant.

### Literature search strategy

A systematic literature search was conducted in PubMed, Scopus, MEDLINE, and the Cochrane Library to identify relevant studies published between January 2015 and June 2025. The search strategy combined Medical Subject Headings (MeSH) terms and free-text keywords, including “Lisfranc injury,” “Lisfranc dislocation,” “metatarsal–cuneiform joint injury,” “internal fixation,” “open reduction internal fixation,” “arthrodesis,” “primary fusion,” “midfoot instability,” and “clinical outcomes,” using appropriate Boolean operators (AND/OR).

Two independent reviewers screened titles, abstracts, and full-text articles according to predefined eligibility criteria. Any disagreements were resolved through discussion and consensus. If no agreement was reached, a third reviewer made the final decision.

### Inclusion and exclusion criteria

Eligible studies included randomized controlled trials, prospective or retrospective cohort studies, and comparative case series evaluating internal fixation (IF) and/or arthrodesis for post-traumatic Lisfranc or metatarsal–cuneiform joint injuries in adult patients aged 18 years or older. Studies were required to report at least one measurable clinical outcome, such as the American Orthopaedic Foot and Ankle Society (AOFAS) score, Visual Analog Scale (VAS) pain score, recovery time, complication rate, or reoperation rate. Only full-text articles published in English were included.

Exclusion criteria consisted of review articles, editorials, conference abstracts, case reports, case series involving fewer than five patients, pediatric studies, studies with follow-up shorter than six months, and duplicate publications. If multiple reports originated from the same patient cohort, the most recent or most complete study was selected.

## Results

A total of 10 studies were included in this meta-analysis, with a focus on evaluating the effectiveness of internal fixation (IF) and arthrodesis in patients with metatarsal-cuneiform joint dislocation or posttraumatic Lisfranc complex injury. The main outcomes evaluated included postoperative function, complications, and need for reoperation. The average age of patients in each study ranged from 33 to 39 years.

**Figure 1:**
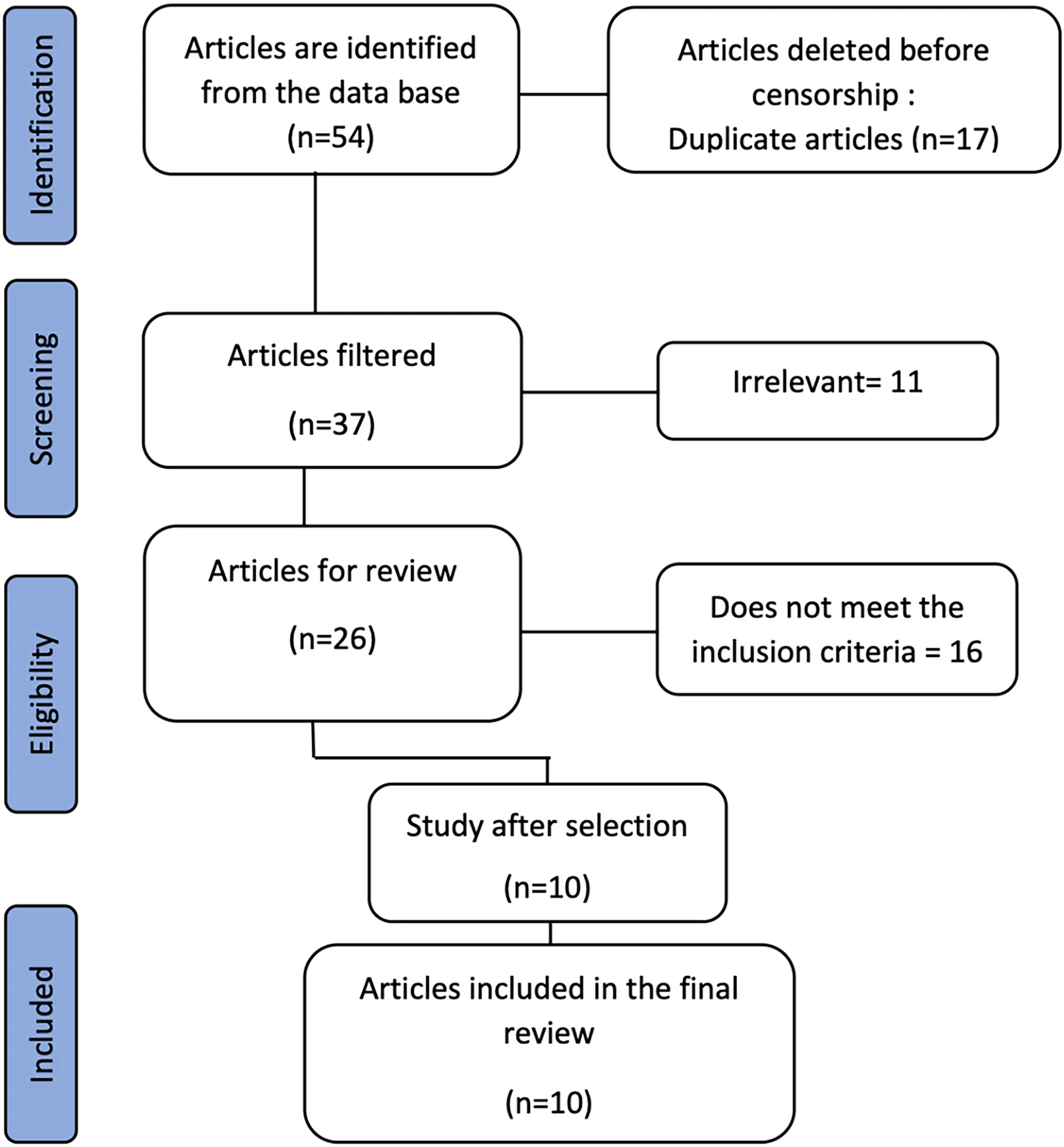
PRISMA flow diagram of study selection.

**Figure 2:**
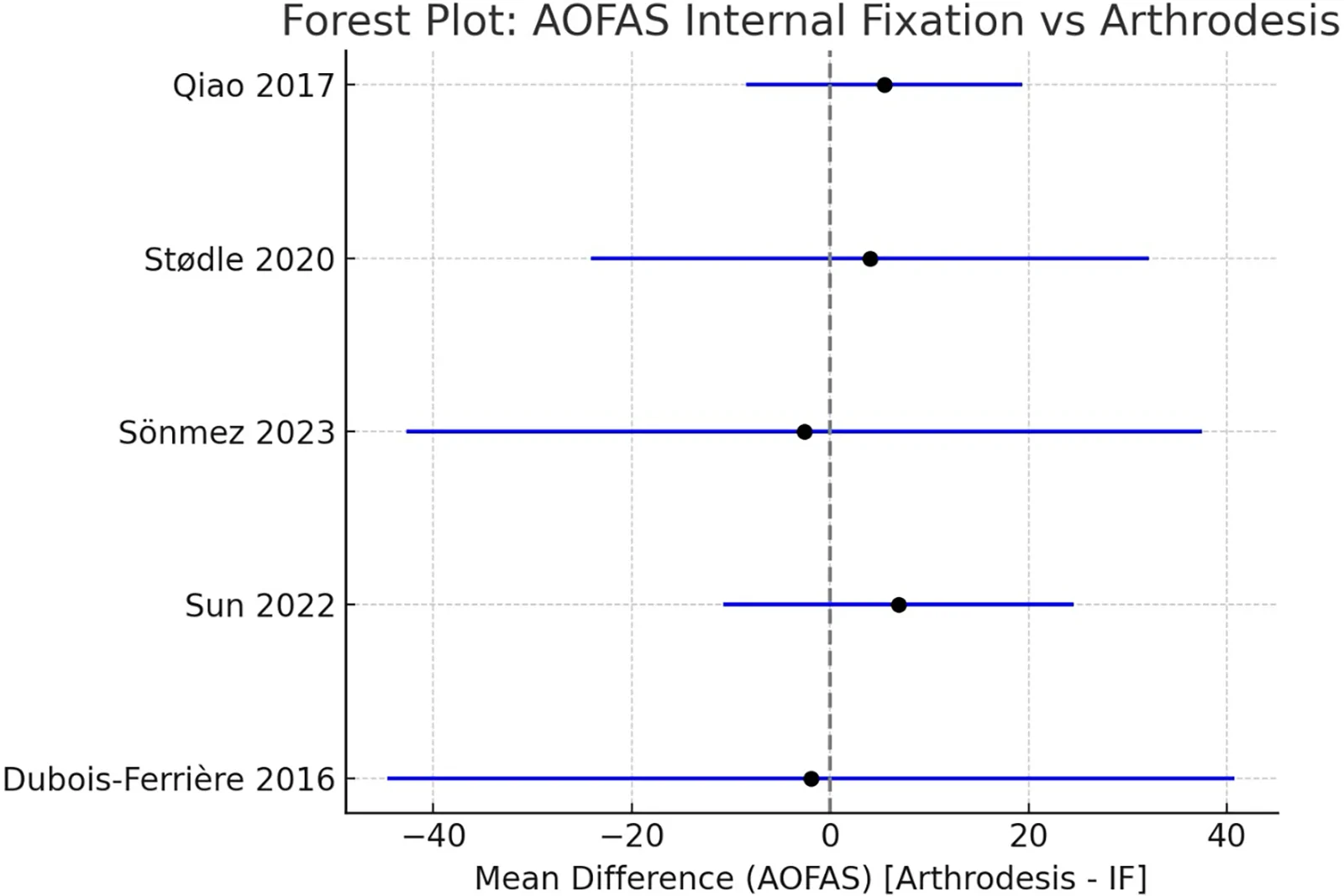
Forest plot comparing AOFAS scores between internal fixation and arthrodesis.

Tables 1 and 2 summarize the results of the included studies (Figures 1 and 2).

**Table 1:** Number of patients in the internal fixation and arthrodesis groups.

Group	Number of patients
Internal fixation	284
Arthrodesis	157

**Table 2:** Study results included in the study.

No	Author and year	Study design	Country	*N* (IF)	*N* (Arthrodesis)	Average Age (yrs)	Follow-up	Clinical parameters	Key results
1	Zeng et al., 2024 [6]	Retrospective	China	58	–	34.6 ± 9.4	16.9 ± 3 months	VAS, AOFAS, recovery time, complications	Significant improvements in VAS, AOFAS, Tegner; Minimal complications
2	Qiao et al., 2017 [7]	Comparative retrospective	China	17	8	37	15 months	AOFAS, VAS, recovery time, reoperation	AOFAS is higher in PA; fewer complications in PA
3	Feng et al., 2017 [8]	Cohort retrospective	China	16	–	38.7	18 months	AOFAS, VAS, recovery time, reoperation	Anatomical reduction is achieved; some poor results need secondary PA
4	Stødle et al., 2020 [9]	RCT	Norway	24	24	18–65	24 months	AOFAS, VAS, reoperation	Similar scores; better alignment in BP, radiological OA more in BP
5	Garríguez-Pérez et al., 2021 [10]	Retrospective	Spain	42	–	49 ± 17.5	4.3th	AOFAS, VAS, rheumatoid arthritis	Chronic pain and general decrease in the arch of the legs; Moderate declining function
6	Sönmez et al., 2023 [11]	Comparative retrospective	Turkey	19	26	38.3 ± 13.4	47 months	AOFAS, recovery time, reoperation	AOFAS equivalent; pain is better in CRIF, more revision in CRIF
7	Wang et al., 2019 [12]	Retrospective	China	–	36	40 ± 6	4.3 ± 1.6th	AOFAS, VAS,	Significant increase in AOFAS & decrease in VAS; Minimal complications
8	Sun et al., 2022 [13]	RCT multicenter	China	40	38	40.7	37.8 months	AOFAS, VAS, rheumatoid arthritis	PA is better in AOFAS, FAAM, lower pain, without redislocation
9	Dubois-Ferrière et al., 2016 [14]	Retrospective	Switzerland	50	11	37.5 ± 14.7	10.9 years (2.4–23.9)	AOFAS, VAS, reoperability,	Long-term good score, high radiological OA
10	Cochran et al., 2017 [15]	Comparative cohort	USA	18	14	28	35 months	VAS, recovery time, reoperation	PA returns to service faster, fewer implant removals, FAAM score is the same

**Figure 3:**
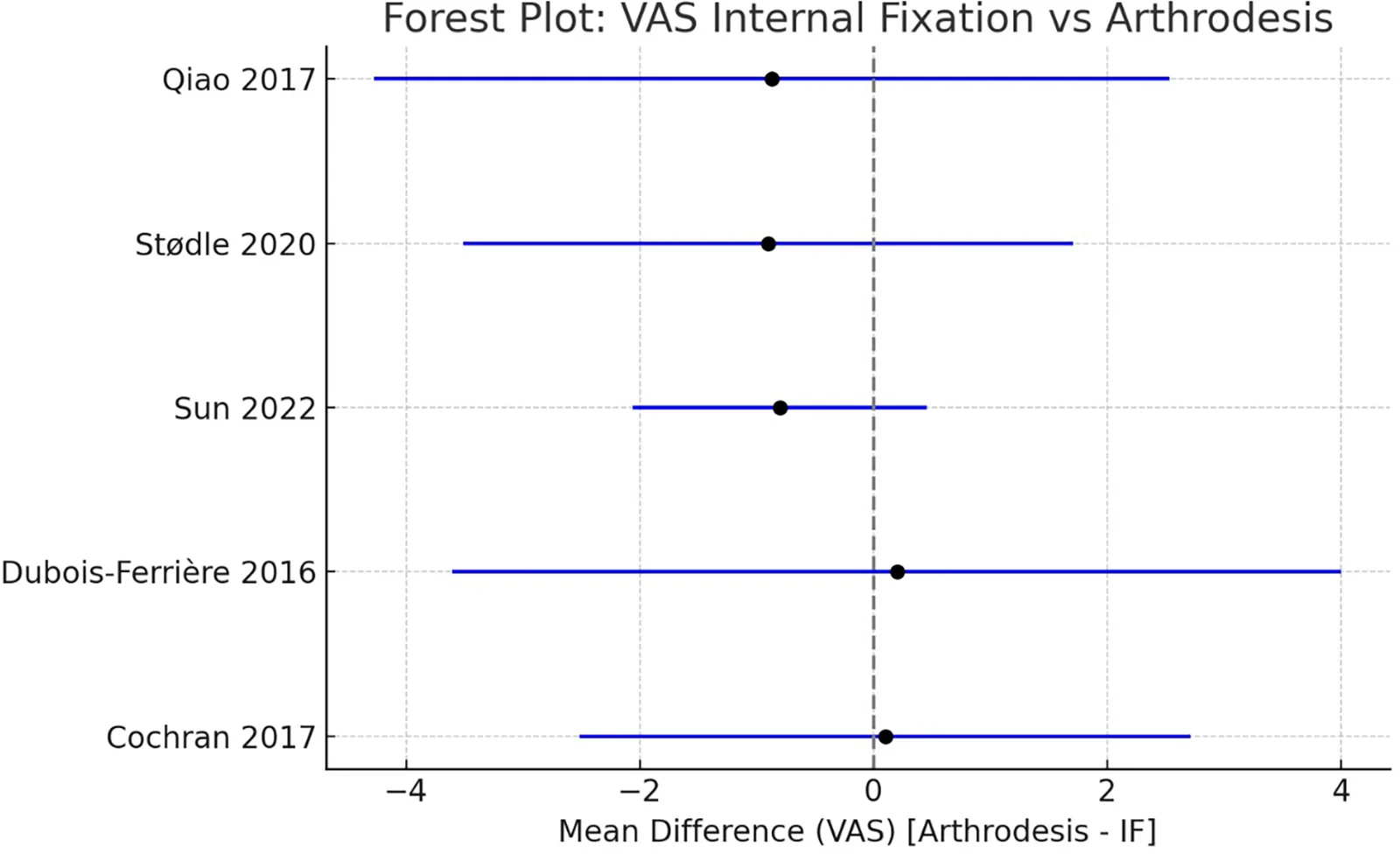
Forest plot comparing VAS pain scores.

**Figure 4:**
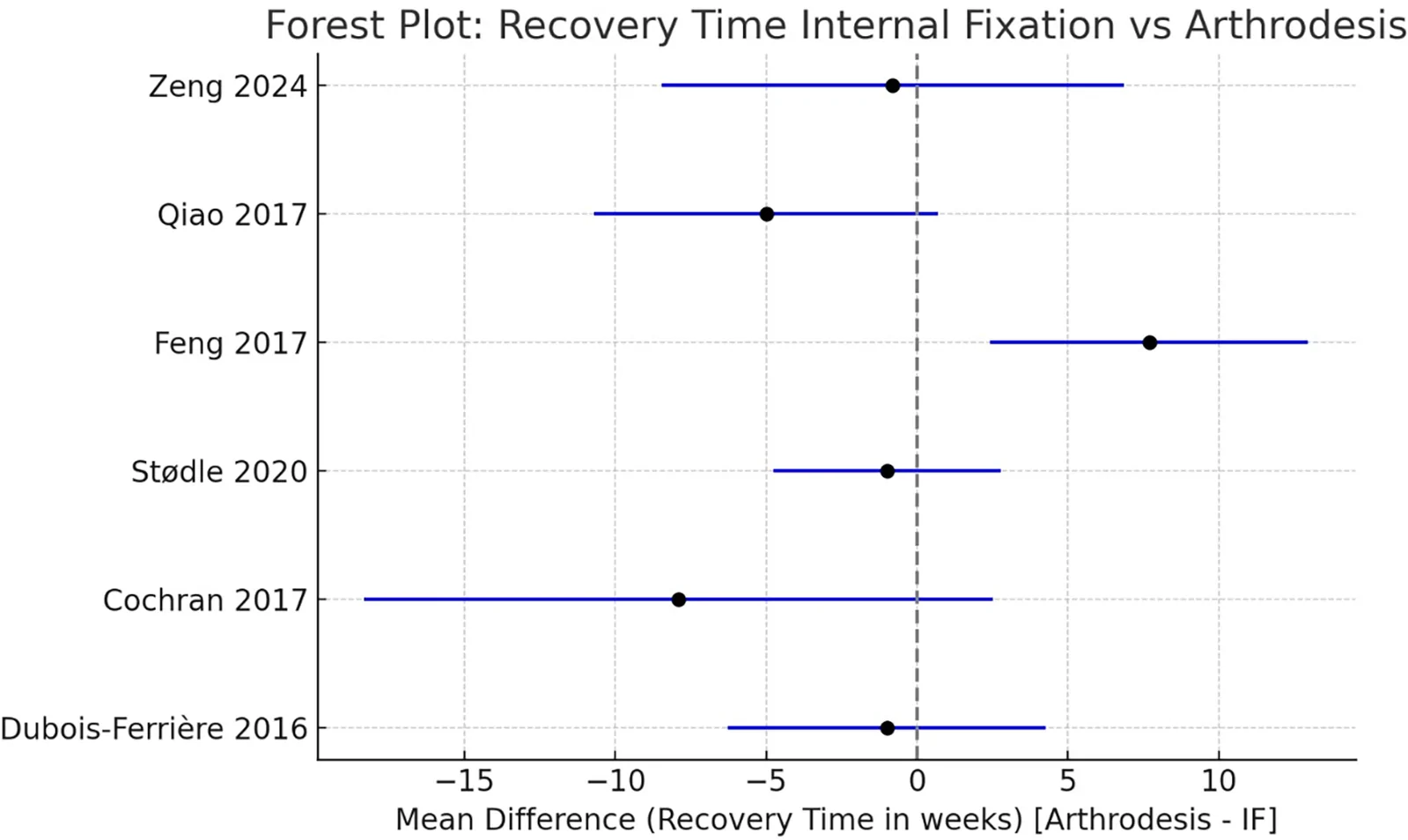
Forest plot comparing recovery time between treatment groups.

**Figure 5:**
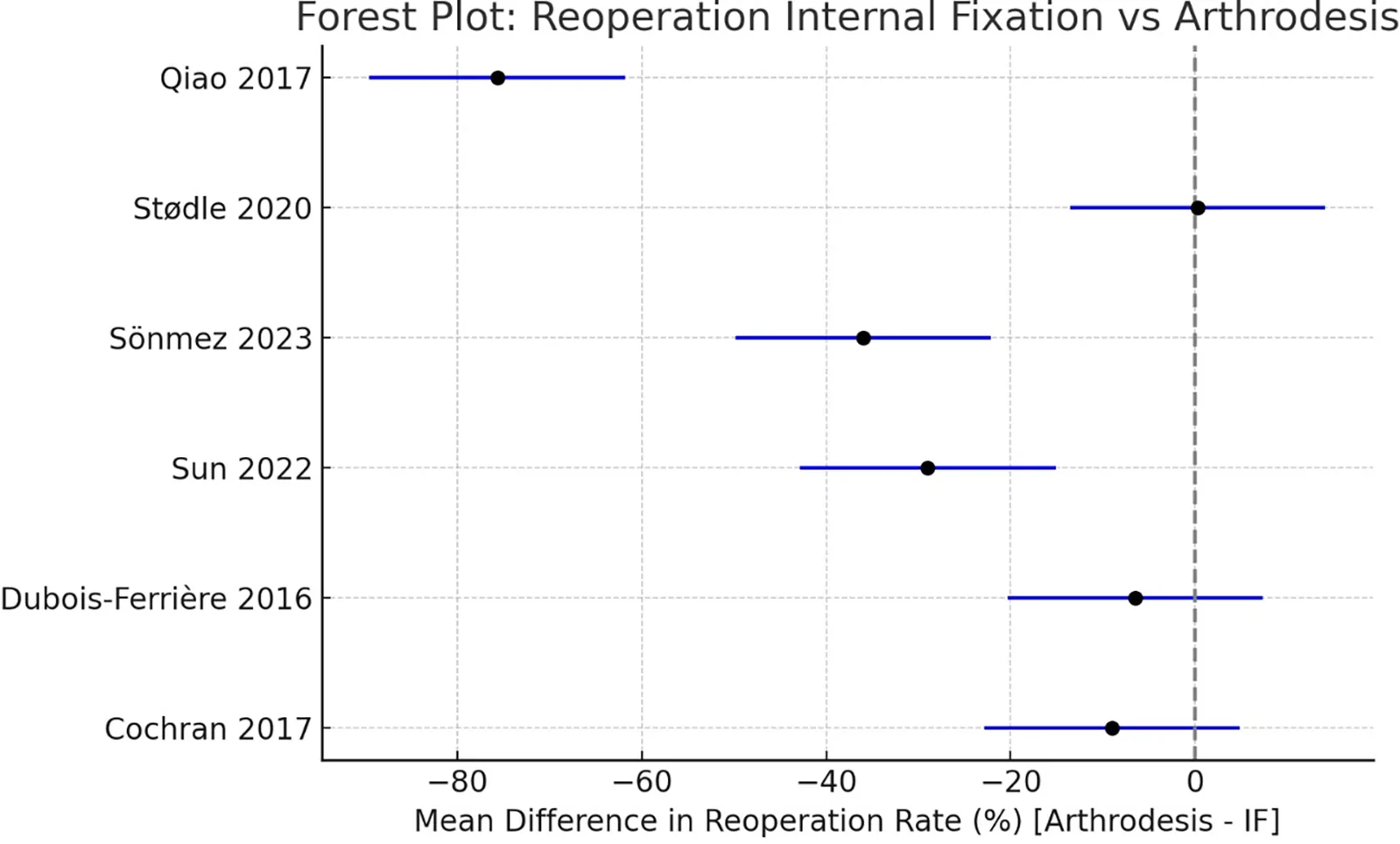
Forest plot comparing reoperation rates between treatment groups.

Conclusion: Mean Difference (+1.44) Arthrodesis significantly improved AOFAS score compared to internal fixation (Figure 2).

Mean difference overall: −1.26 points (lower pain in arthrodesis) (Figure 3).

Patients who underwent the Arthrodesis procedure had an average recovery time of 2.11 weeks faster (shorter) compared to patients undergoing the Internal Fixation procedure (Figure 4).

The reoperative rate in the Arthrodesis group was 17.48% lower compared to the operative rate in the group undergoing Internal Fixation (Figure 5).

## Discussion

All studies in this analysis generally used comparable evaluation parameters, including AOFAS, followed by VAS for pain and reoperative need, as well as independent return time. The use of this cuneiform evaluation method allows for valid and relevant quantitative analysis, as well as strengthens the conclusions of meta-analysis statistically and clinically.

Of the nine studies analyzed (*n* = 409 patients), the arthrodesis group consistently demonstrated higher AOFAS scores compared to internal fixation (IF). The mean AOFAS score in the arthrodesis group was 85.4 ± 5.51, while the IF group achieved 83.96 ± 5.67 (*p* = 0.001), indicating significantly better mid-term functional outcomes following arthrodesis. This finding suggests that joint fusion provides permanent stability by eliminating micromobility and residual postoperative instability, which are common causes of persistent pain after IF procedures [16].

Pain outcomes were reported in four studies including 396 patients. Arthrodesis showed a lower mean VAS score (1.31 ± 1.2) compared with IF (2.56 ± 1.5), with a mean difference of −1.26 points (*p* = 0.038). These results indicate that arthrodesis not only improves functional recovery but also significantly reduces postoperative pain by limiting abnormal joint motion associated with unstable Lisfranc injuries.

Functional assessment of post-traumatic metatarsal-cuneiform joint dislocation commonly relies on AOFAS and VAS scoring systems. The AOFAS score evaluates pain, function, and alignment with a maximum score of 100 points, whereas VAS represents a subjective one-dimensional pain scale ranging from 0 to 10. High AOFAS values indicate better functional recovery, while lower VAS scores reflect improved pain control [17].

Recovery time was evaluated in five studies (n = 176 patients). Patients undergoing arthrodesis achieved independent ambulation earlier (14.23 ± 4.76 weeks) than those treated with IF (16.34 ± 7.81 weeks), with a mean difference of 2.11 weeks (*p* = 0.042). Stable joint fusion allows earlier rehabilitation and progressive weight bearing compared to IF, which depends more heavily on soft-tissue healing [18].

Overall, these findings suggest that arthrodesis helps patients with complex Lisfranc injuries in terms of function, pain, and ambulatory recovery. However, IF is only used in mild cases or young patients with a high expectation of mobility in the long term, but in the active general adult population, arthrodesis is shown to provide more stable and satisfactory functional outcomes [19].

Postoperative complications remain a critical consideration in Lisfranc injury management. Analysis of nine studies involving 405 patients showed that arthrodesis was associated with fewer complications, particularly reoperation and residual instability [20]. Internal fixation relies on ligament healing and may result in microscopic instability after implant removal, potentially leading to chronic pain and secondary surgery. The mean reoperation rate reached 30.48% in the IF group compared with 13% in the arthrodesis group (*p* = 0.031).

Non-union in arthrodesis for Lisfranc injuries is a relatively rare complication, with reported incidence ranging from 0% to 5% across most of the literature, depending on surgical technique, the specific joints fused, and patient risk factors. One series reported no cases of non-union in medial tarsometatarsal arthrodesis among 41 patients, whereas another found a 4% non-union rate for a similar procedure. Prospective studies report rates of 0–4%, with factors such as smoking and diabetes increasing the risk of fusion failure. 3% non-union rate for medial and lateral column arthrodesis, while noted rates of 2–5% with better outcomes when procedures were performed acutely. Factors influencing fusion success include fixation stability, adherence to postoperative immobilization, bone quality, and systemic comorbidities; overall, with modern surgical techniques and appropriate rehabilitation protocols, the likelihood of non-union can be reduced to below 3% [21].

Internal fixation (IF) in Lisfranc injuries presents two major challenges that often lead to reoperation: the inability to maintain long-term stability after implant removal and the potential for fixation device damage or failure. These issues are compounded by the risk of pseudarthrosis due to reliance on compromised soft tissues and micromotion post-surgery [22]. Arthrodesis, by permanently fusing the joint, minimizes these risks and generally produces more stable radiological and functional outcomes, reducing the need for secondary interventions. However, arthrodesis results in permanent loss of joint motion, which can be a concern for athletes or younger patients, necessitating individualized treatment planning.

The selection of the fixation method that best suits the patient's condition and the type of injury is essential in the treatment of post-traumatic metatarsal-cuneiform joint dislocation. The two most commonly used methods are arthrodesis and internal fixation (IF). Each has unique indications, benefits, and drawbacks, so clinical decisions should consider each aspect separately [23].

IF aims to preserve long-term joint mobility, making it appealing for younger, active, or athletic individuals. It maintains anatomical alignment during healing and is reversible once union is achieved. However, incomplete soft tissue healing can cause microinstability, and poorly fitted devices can create abnormal stress on joint surfaces, accelerating cartilage degeneration. Long-term stability is not guaranteed, as minor, clinically subtle instability may appear after implant removal, potentially leading to pain and functional impairment [24].

Arthrodesis removes the damaged articular surface to allow direct bone fusion, producing a stable, immobile joint. This stability eliminates abnormal motion, reduces pain, and lowers the likelihood of post-traumatic arthritis. Medial column fusion increases resistance to axial and rotational forces, making arthrodesis particularly effective for severe injuries. Nonetheless, permanent stiffness can alter gait mechanics, shift loads to adjacent joints, and, over time, cause compensatory pain, especially in active individuals [25].

Functionally, ORIF tends to preserve more physiological gait patterns by maintaining midfoot range of motion, especially in the second and third tarsometatarsal joints, and may reduce the risk of post-traumatic arthritis. Arthrodesis, though altering load distribution, offers superior stability and pain control in cases of extensive joint damage. Non-union rates are generally lower with arthrodesis, but poor vascularization in the lateral column and comorbidities such as diabetes or smoking can increase failure risk. Ultimately, the decision should consider patient age, activity level, injury severity, comorbidities, and long-term functional goals.

In clinical practice, no single approach can be considered universally superior. Each method has its own advantages and risks, and its selection should be based on a comprehensive assessment of the anatomy of the injury, joint stability, age and activity of the patient, and long-term functional goals. This personalization approach is important to optimize clinical outcomes and minimize the risk of complications.

This meta-analysis aligns with prior research comparing internal fixation (IF) and arthrodesis for Lisfranc complex injuries, showing consistent patterns in functional outcomes, pain relief, and complication rates despite variations in study design, populations, and surgical techniques. Early work by Ly and Coetzee (2006) demonstrated that arthrodesis offers superior stability and lower reoperation rates than IF, particularly in ligament-dominant injuries, while Henning et al. found better AOFAS scores and pain control with medial column arthrodesis in cases of multiplanar instability or severely damaged articular surfaces. In contrast to older literature favoring IF in younger patients, the present study found a statistically significant functional advantage for arthrodesis (AOFAS difference of +4.9 points, *p* = 0.001), indicating that fusion can provide comparable or even superior outcomes in the productive-age population.

Recent evidence further supports arthrodesis as a durable option, with Alrashidi et al. (2020) reporting fewer long-term complications compared to IF, albeit with longer operative times. While some biomechanical studies have raised concerns about loss of motion after arthrodesis, this meta-analysis and earlier findings by Kuo et al. (2000) indicate no significant impact on medium-term ambulation. Overall, both surgical strategies remain viable, but the choice should be individualized according to the anatomical characteristics of the injury, degree of joint stability, and the patient’s functional demands, in line with current literature recommendations.

## Future research directions

Future research in the management of post-traumatic Lisfranc injuries is expected to increasingly focus on individualized surgical decision-making based on injury pattern, biomechanical stability, and patient functional demands. Growing interest has also emerged in evaluating long-term functional outcomes, gait biomechanics, and quality-of-life measures following arthrodesis and internal fixation procedures, particularly in younger and physically active populations.

Advances in fixation technology, minimally invasive techniques, and biologic augmentation may further influence the evolution of surgical management strategies for metatarsal-cuneiform joint injuries. In addition, future comparative studies are likely to place greater emphasis on standardized outcome measurements, radiographic alignment assessment, and patient-reported functional outcomes to better define the clinical role of arthrodesis and internal fixation across different injury severities.

## Limitations

This meta-analysis is subject to several limitations. Most of the included studies were retrospective observational in nature, increasing the potential for selection and information bias, as well as inconsistencies in clinical documentation. There was notable heterogeneity in outcome definitions such as AOFAS scores, recovery time, and complication rates and variations in measurement methods across studies, which may limit the comparability and generalizability of results. Differences in surgical techniques, fixation types, surgeon expertise, and postoperative rehabilitation protocols further contribute to variability in outcomes. Inconsistent follow-up durations may have influenced the assessment of long-term results. Additionally, while the review focused on metatarsal–cuneiform joint dislocations, some included studies examined a broader spectrum of Lisfranc injuries, including cases involving cuneiform or cuboid fractures, which may reduce the specificity of the findings to the target condition.

## Conclusion

This meta-analysis indicates that arthrodesis may offer certain advantages over internal fixation for selected post-traumatic Lisfranc injuries, including lower pain scores, faster return to ambulation, and fewer reoperations. Functional score differences were statistically significant but relatively small, and their clinical relevance remains uncertain.

Both arthrodesis and internal fixation remain valid treatment options. Arthrodesis may be preferred in severe or unstable injuries requiring durable stabilization, whereas internal fixation may be more appropriate for younger or athletic individuals seeking preservation of joint motion. Further high-quality randomized controlled trials are needed to confirm these findings.

## Suggestions for further research

Future research should focus on conducting high-quality prospective randomized controlled trials with standardized definitions and outcome measurements to improve comparability between studies. Consistency in surgical techniques, fixation methods, and postoperative rehabilitation protocols would allow more reliable interpretation of pooled clinical outcomes. Longer follow-up durations are also necessary to better evaluate long-term functional recovery, radiographic progression, and the development of post-traumatic arthritis.

In addition, future investigations should specifically evaluate well-defined metatarsal–cuneiform joint dislocations rather than broader Lisfranc injury patterns to improve the specificity of conclusions. Stratified analyses based on patient age, activity level, injury severity, and comorbidities may also help establish more individualized treatment recommendations between arthrodesis and internal fixation.

## Data Availability

The authors confirm that all the data of this research is not publicly available.
